# Atlantic City, N. J., September 3, 4, and 5, 1901

**Published:** 1900-12

**Authors:** 


ATLANTIC CITY, N. J., SEPTEMBER 3, 4, AND 5, 1901.

President Butler, of the Association, has just announced the following Committee of Arrangements: Dr. William Herbert Lowe, 188-190 Ellison Street, Paterson, N. J., Chairman. Drs. T. Earle Budd, Orange; T. E. Smith, Jersey City, N. J.; Robert W. Ellis, New York City, N. Y., and W. Horace Hoskins, Philadelphia, Pa.

President S. J. J. Harger, of the Pennsylvania State Veterinary Medical Association, has just announced the following committee of that organization to co-operate with the profession in New Jersey in promoting the success of the Atlantic City meeting—Drs. Leonard Pearson, C. J. Marshall, W. Horace Hoskins, and Francis Bridge. President Harger will personally direct the efforts of the Keystone State Committee.

The newly elected President of the Keystone Veterinary Medical Association, Dr. J. D. Houldsworth, will shortly announce a committee of that body to aid the veterinarians of New Jersey in completing the programme of entertainment of those who will visit New Jersey's Mecca by the Sea in September, 1901.

Treasurer Lowe, of the A. V. M. A., says the Atlantic City meeting in his own State will be the first meeting of the new century, and it must rival or excel any meeting of its kind on this continent. Every one who knows Lowe knows him to be a hustler.

The annual meeting of the Veterinary Medical Association of New Jersey, which will be held in Trenton on January 10th, will enter thoroughly upon the work of preparation for the coming meeting of the A. V. M. A. at Atlantic City.

A special effort will be made to have the surgical clinics occupy a half day and to be conducted upon a high plane in thorough keeping with the advances all along the lines of surgery.

Maryland should take an active interest in the Atlantic City meeting. Resident State Secretary Nolan, of that State, should call together the stalwart supporters of the A. V. M. A., and plan for a strong interest in behalf of Maryland's strong arm of the profession. Dr. Dougherty would fittingly direct her interests as chairman of such a body.

Pennsylvania will not wait for her meeting in March to get in line to assist the meeting at Atlantic City. President Harger has already taken action. President Houldsworth will no doubt ask a representative of Delaware to aid the committee from the Keystone Veterinary Medical Association. The JOURNAL suggests the name of Dr. Thomas B. Rayner as chairman of the Keystone representatives. His almost constant attendance at the meeting in other cities for ten years makes his services valuable in advice and effective work.

September in Atlantic City is one of the most delightful periods of the summer season. The rush of July and August are past, and while the city is in gala attire, the hotels in friendly rivalry over the entertainment of their guests, the attractions of the boardwalk undimmed, the comforts to be obtained are so much better than at any other period of the year.

The Amalgamated Associations of New Jersey will appoint a strong committee to direct the affairs incidental to the coming meeting, and will invoke the aid of a large contingent of the South Jersey veterinarians.

Early in November several veterinarians from Washington, D. C., called at the JOURNAL office to express their favor of Atlantic City for 1901.

Those visiting the Pan-American Exposition in Buffalo on their way to Atlantic City will have the opportunity of viewing a milking machine, built on the pneumatic system, with valves, suction rubbers, etc. It is exhibited by a Scottish firm.

The Burlington Route, that has contributed so much to the success of a number of the meetings in the West during the past five years, will make a special effort to send from the great Western centres of this railroad large delegations to Atlantic City *via* Buffalo and Niagara Falls. The uniform courtesy of the officials of this extensive route will ever be remembered with gratitude by the members of the A. V. M. A.

